# Supplementary material for: A nuclear receptor HR96-related gene underlies large trans-driven differences in detoxification gene expression in a generalist herbivore
Source: Nat Commun. 2023 Aug 17;14:4990. doi: 10.1038/s41467-023-40778-w (PMC10435515; doi:10.1038/s41467-023-40778-w)

A nuclear receptor HR96-related gene underlies large *trans*-driven differences in detoxification gene expression in a generalist herbivore

Meiyuan Ji[1,†], Marilou Vandenhole[2,†], Berdien De Beer[2,†], Sander De Rouck[2], Ernesto Villacis-Perez[2], René Feyereisen[2], Richard M Clark[1,3,*] and Thomas Van Leeuwen[2,*]

[1]School of Biological Sciences, University of Utah, Salt Lake City, UT 84112, USA

[2]Department of Plants and Crops, Faculty of Bioscience Engineering, Ghent University, Ghent, Belgium

[3]Henry Eyring Center for Cell and Genome Science, University of Utah, Salt Lake City, UT 84112, USA

[†]Equal contribution

*Corresponding

Supplementary Information

Supplementary Figure 1: Non-random recombination across *T. urticae* chromosomes.

Supplementary Figure 2: Schematic demonstrating the assignment of genotype bins for use in eQTL mapping.

Supplementary Figure 3: Most local eQTLs cluster within several hundred kb of their associated genes.

Supplementary Figure 4: Negative log-transformed adjusted-*p* values and absolute values of effect sizes for *cis* and *trans* eQTLs associated with all genes and detoxification genes.

Supplementary Figure 5: Effect size distributions for *trans* eQTLs in *trans* eQTL hotspots.

Supplementary Figure 6: The HS5 *trans* eQTL hotspot co-localizes with a peak of deviation in the proportion of the RS genotype in the eQTL mapping population.

Supplementary Figure 7: Genome-wide genotypic characterizations of A-NIL-HS1^{RR}, A-NIL-HS1^{SS}, B-NIL-HS1^{RR}, and B-NIL-HS1^{SS}.

Supplementary Figure 8: Genomic structure of the *HR96-LBD-1a* and *HR96-LBD-1b* region in the R, S, and C1N1d strains.

Supplementary Figure 9: Alignment of HR96-LBD-1a and HR96-LBD-1b sequences from the R and S strains.

Supplementary Figure 10: Expression of *tetur86g00030* on bean and tomato following injections with dsGFP and dsHR96-LBD-1.

Supplementary Figure 11: The W309R strain variant in HR96-LBD-1b is predicted to reside in the ligand-binding pocket.

Supplementary Figure 12: Nucleotide variation in *T. urticae* strains resulting in the W309R amino acid change.

Supplementary Figure 1: Non-random recombination across *T. urticae* chromosomes.

For *T. urticae* chromosomes 1-3, cumulative deviations from the expected number of recombination events in non-overlapping 1.5 Mb windows, assuming no biases in recombination (random assignment), were assessed 50,000 times (x-axis, with frequency shown on the y-axis); the expected number of recombination events per window was based on the observed number of recombination events per chromosome as detected by genotyping of the 458 F3 families divided by the number of bins per chromosome. Dashed blue vertical lines denote the empirical (permutation-established) *p*-value cutoff of 0.001 by chromosome (a one-sided test with the alternative hypothesis being non-random recombination). The observed values (vertical dashed red lines) for each of the three chromosomes all fall to the right of the blue dashed lines (for each chromosome, the observed values are more extreme than any of those in the distributions established by permutation).

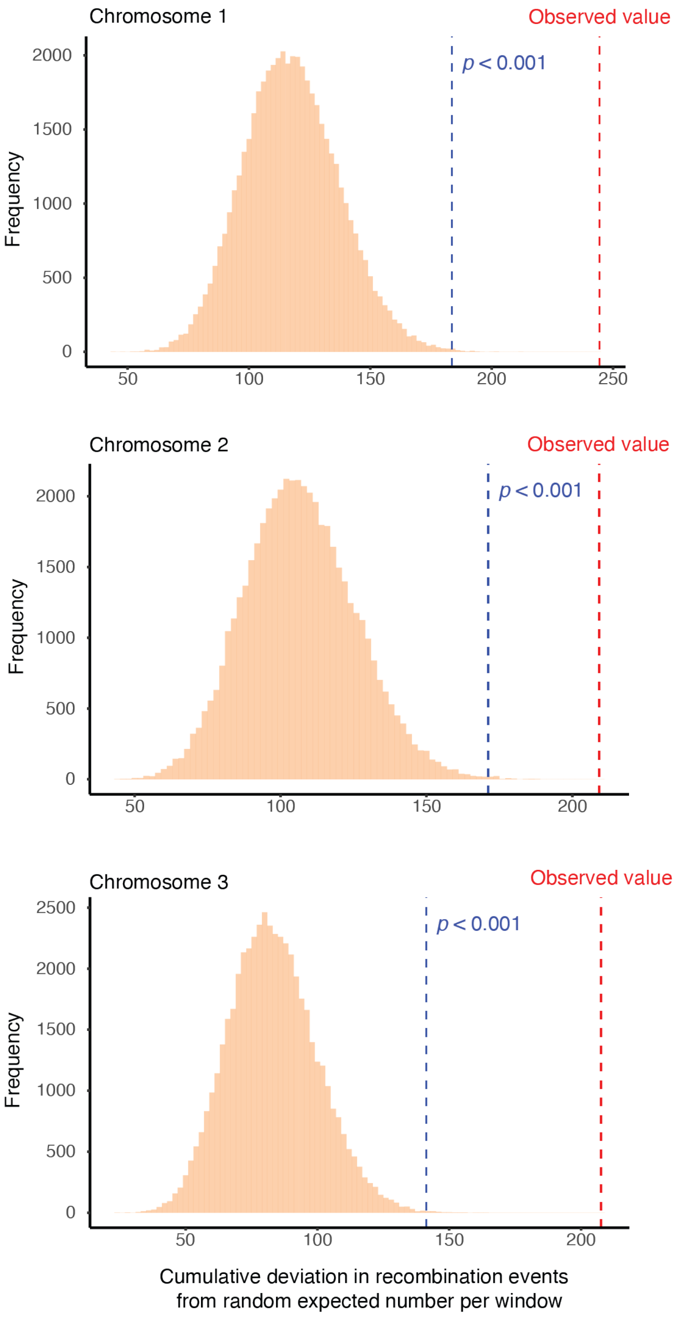

Supplementary Figure 2: Schematic demonstrating the assignment of genotype bins for use in eQTL mapping.

Shown in white or gray shading are genotype blocks (legend, top right) for a subset of the 458 F3 isogenic families (Fam. 1-10) that collectively constitute the eQTL mapping population (the specific breakpoints between genotypes are created for illustration only, with the x-axis showing relative position along a chromosome). Given our experimental design, the two possible genotypes at a locus in any given F3 isogenic family are either RS (heterozygosity for the R and S strain haplotypes) or SS (homozygosity for the S strain haplotype). Genotype bins were assigned as the intervals between unique recombination events as assessed across all families (vertical dashed red lines); where two recombination events occurred at the same location, the events were collapsed to a single event for genotype bin construction.

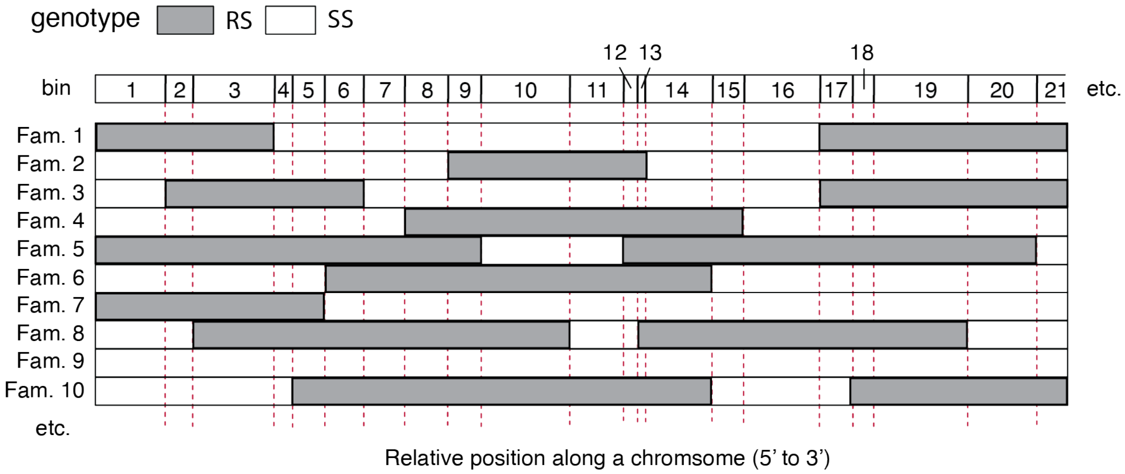

Supplementary Figure 3: Most local eQTLs cluster within several hundred kb of their associated genes.

Histogram of distances for eQTLs to their associated genes where distances are within ±1.5 Mb. The dashed vertical lines at ±800 kb denote the cutoff used for *cis* eQTL assignment.

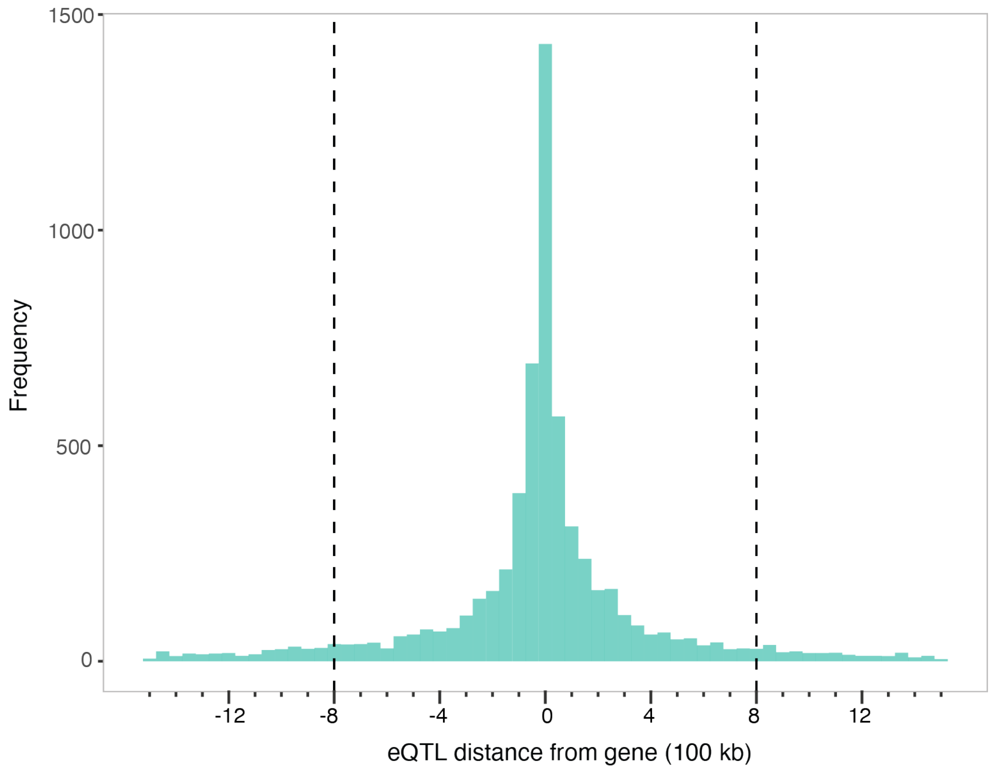

Supplementary Figure 4: Negative log-transformed adjusted-*p* values and absolute values of effect sizes for *cis* and *trans* eQTLs associated with all genes and detoxification genes.

The $-\log_{10}$(adj-*p*) values for *cis* and *trans* eQTLs associated with all genes and detoxification genes are shown, top left and right, respectively ; absolute effect size values (evaluated using a linear model) for *cis* and *trans* eQTLs for all genes and detoxification genes are shown, bottom left and right, respectively (for display, a combination of violin and boxplots are used; the adjusted *p*-values and effect sizes are from the output of MatrixEQTL). For *cis* and *trans* eQTLs, the number of associations is indicated below the x-axis (*n*). Significant differences in $-\log_{10}$(adj-*p*) or absolute effect size values between *cis* and *trans* eQTLs for each of the four comparisons were detected (two-sided Wilcoxon rank sum tests, all $p < 10^{-15}$). For the boxplots, box indentations are median values, boxes extend from the first to third quartiles, whiskers extend to 1.5 times the respective interquartile ranges, and outliers are plotted. Data upon which the figure is based are provided in Supplementary Data 3.

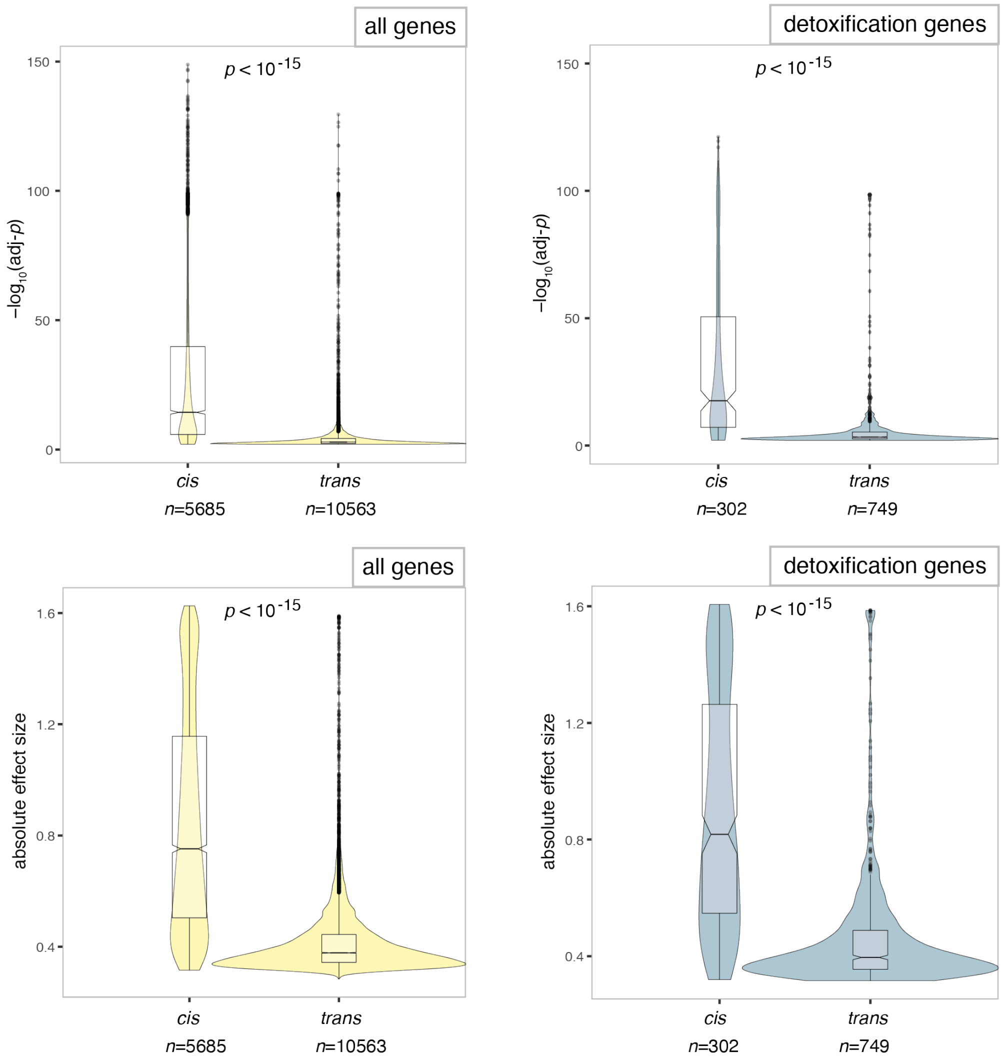

Supplementary Figure 5: Effect size distributions for *trans* eQTLs in *trans* eQTL hotspots.

Effect sizes of all *trans* eQTLs in hotspots, as assessed with MatrixEQTL, are given for each of HS1-HS9. Positive values reflect instances where the RS genotype at an eQTL (heterozygosity for both the R and S strain haplotypes) resulted in higher expression of the target gene as compared to the SS genotype (homozygosity for the S strain haplotype). Negative values reflect the opposite. The number of associations (*n*) for each *trans* eQTL hotspot are shown at the top. The distributions are displayed with a combination of violin and boxplots. For the boxplots, box indentations are median values, boxes extend from the first to third quartiles, whiskers extend to 1.5 times the respective interquartile ranges, and outliers are plotted. Data upon which the figure is based are provided in Supplementary Data 6. For the nine *trans* eQTL hotspots, where a significant bias (adj-$p < 0.05$, denoted by an asterisk) in the direction of effect conferred by *trans* regulation was observed, "up" or "down" is noted (for HS1-HS9, respectively, chi-square goodness of fit test results are: $\chi^2(1) = 12.66$, $p = 0.003$; $\chi^2(1) = 8.37$, $p = 0.035$; $\chi^2(1) = 18.00$, $p = 1.99 \times 10^{-4}$; $\chi^2(1) = 7.35$, $p = 0.060$; $\chi^2(1) = 38.09$, $p = 6.09 \times 10^{-09}$; $\chi^2(1) = 26.88$, $p = 1.94 \times 10^{-06}$; $\chi^2(1) = 85.38$, $p = 2.21 \times 10^{-19}$; $\chi^2(1) = 77.14$, $p = 1.44 \times 10^{-17}$; $\chi^2(1) = 5.25$, $p = 0.197$; p values adjusted with the Bonferroni method to account for multiple tests).

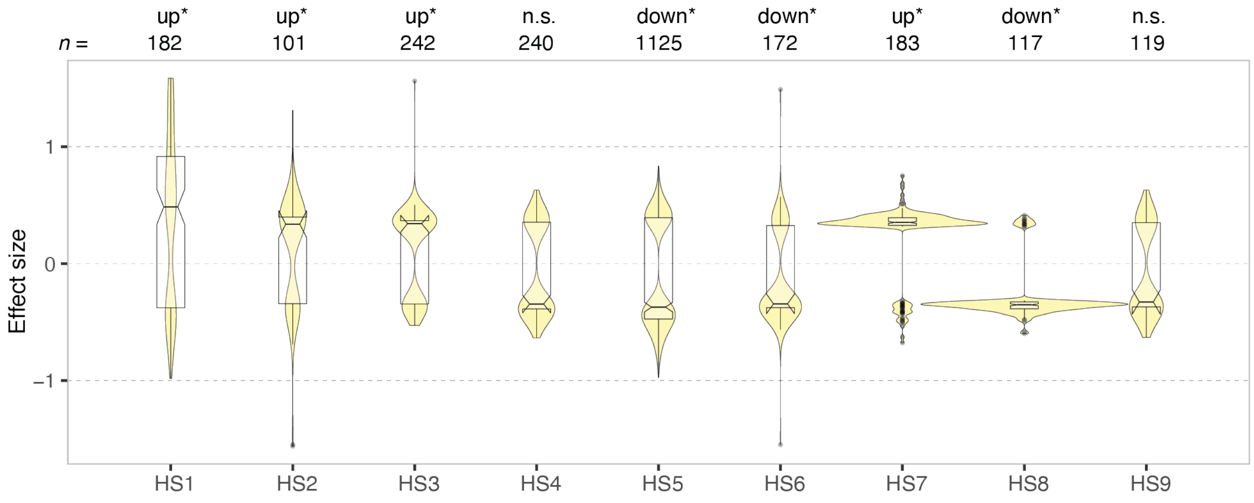

Supplementary Figure 6: The HS5 *trans* eQTL hotspot co-localizes with a peak of deviation in the proportion of the RS genotype in the eQTL mapping population.

Stacked bar plot showing the number of *trans* eQTL in 200 kb windows (see also Fig. 2c). The directional effect was evaluated by comparing expression levels between the heterozygous RS versus homozygous SS genotypes at the respective *trans* eQTL loci in the 458 F3 families (see legend, top). *Trans* eQTL hotspots (HS1-HS9) are denoted for windows with eQTL associated with expression variation of > 100 genes in *trans* (horizontal black dashed line). Overlayed is a line plot showing the proportion of the RS genotype every 50 kb as determined across the mapping population of 458 F3 families (the orange dashed line indicates the expected ratio of 0.5 given the crossing design, see Fig. 1c). For HS1-HS9, where a significant bias (adj-p < 0.05, denoted by an asterisk) in the direction of effect conferred by *trans* regulation was observed, "up" or "down" is given in parentheses (for HS1-HS9, respectively, chi-square goodness of fit test results are: $\chi^2(1) = 12.66$, $p = 0.003$; $\chi^2(1) = 8.37$, $p = 0.035$; $\chi^2(1) = 18.00$, $p = 1.99 \times 10^{-4}$; $\chi^2(1) = 7.35$, $p = 0.060$; $\chi^2(1) = 38.09$, $p = 6.09 \times 10^{-09}$; $\chi^2(1) = 26.88$, $p = 1.94 \times 10^{-06}$; $\chi^2(1) = 85.38$, $p = 2.21 \times 10^{-19}$; $\chi^2(1) = 77.14$, $p = 1.44 \times 10^{-17}$; $\chi^2(1) = 5.25$, $p = 0.197$; p values adjusted with the Bonferroni method to account for multiple tests). This figure is based on the source data for Fig. 1c and Fig. 2c.

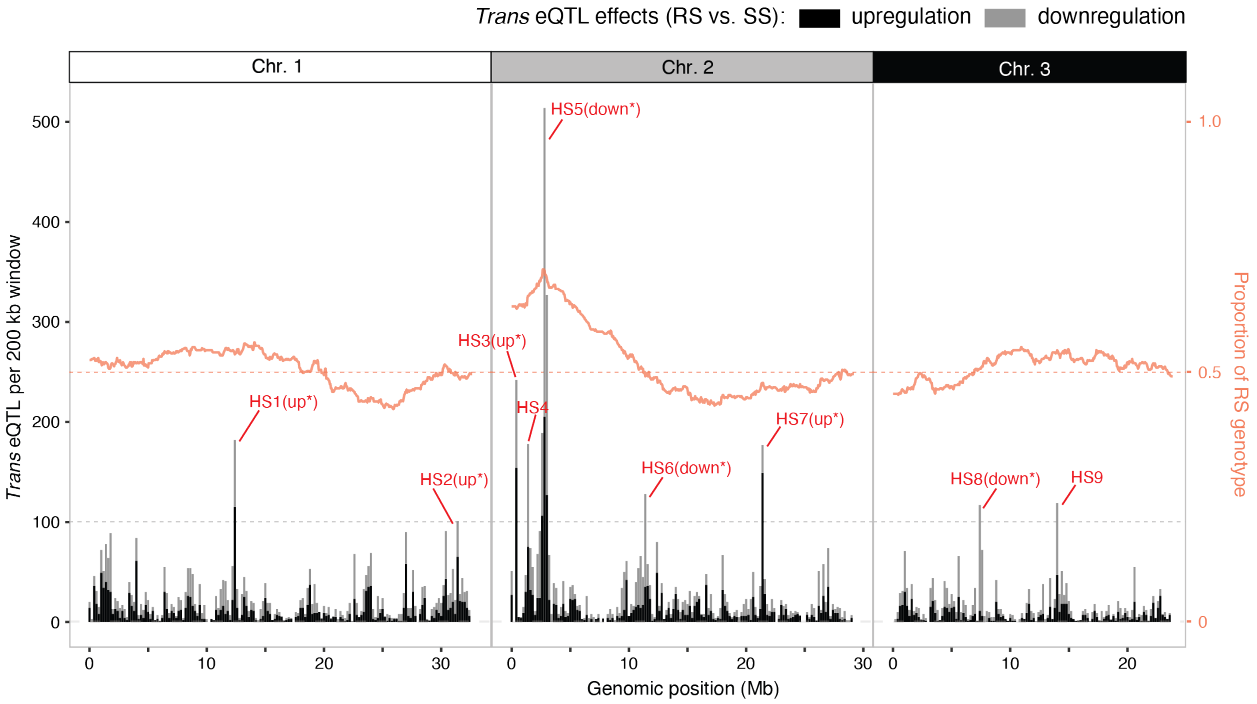

Supplementary Figure 7: Genome-wide genotypic characterizations of A-NIL-HS1^{RR}, A-NIL-HS1^{SS}, B-NIL-HS1^{RR}, and B-NIL-HS1^{SS}.

Genotypes of A-NIL-HS1^{RR} and A-NIL-HS1^{SS} (top left and right, respectively) and B-NIL-HS1^{RR} and B-NIL-HS1^{SS} (bottom left and right, respectively) as determined by RNA-seq-based genotyping. Both A-NIL-HS1^{RR} and B-NIL-HS1^{RR} are homozygous for the R haplotype for small intervals at HS1 (red lettering and arrow) on chromosome 1 at ~12.5 Mb (introgressed R haplotypes of ~530 and ~700 kb, respectively). Otherwise, the SS genotype is present in all NILs except for an ~3 Mb region of segregation (heterozygosity) in B-NIL-HS1^{RR} and B-NIL-HS1^{SS} at the beginning of chromosome 1 as indicated (denoted by shading one chromosome black; the R and S strain haplotypes are as indicated in the legend, bottom). Genotype breakpoints were assigned at the midpoints between SNPs with contrasting genotypes as inferred from RNA-seq read alignments (see Methods).

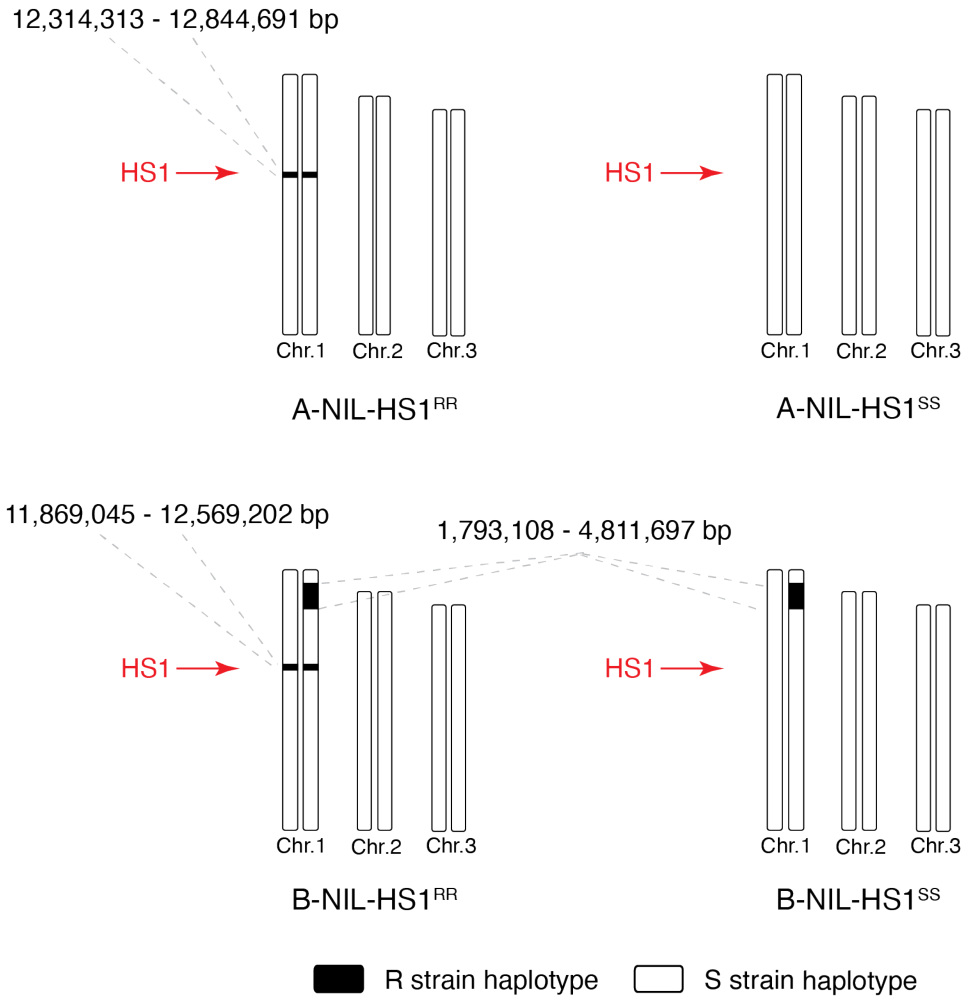

Supplementary Figure 8: Genomic structure of the *HR96-LBD-1a* and *HR96-LBD-1b* region in the R, S, and C1N1d strains.

The genomic structure and positions of *HR96-LBD-1a* and *HR96-LBD-1b* (blue, see legend at bottom right) in the R strain (top panel; contig_78 in the R strain PacBio genome assembly), the S strain (middle panel; scaffold_17 in the S strain PacBio assembly), and the C1N1d strain (bottom panel; scaffold187 in the Illumina C1N1d assembly) are shown. For the R and S strains, *HR96-LBD-1a* and *HR96-LBD-1b* are located as tandem duplicates (direct repeats) on the forward strand of the respective sequences in the two assemblies (the respective annotation coordinates are provided in Supplementary Data 13); for the C1N1d strain, the indicated coordinates for the sequence used for multiple alignment (see Methods) are for the reverse complement of scaffold187. Coding exons of genes are shown as rectangles with a line extending from the first to the last exons in the respective gene models (exon numbers are provided in parentheses underneath), and arrows indicate the direction of transcription. The intergenic distance between the tandemly duplicated *HR96-LBD-1a* and *HR96-LBD-1b* genes in the R strain is 1667 bp, and the respective intergenic distance in the S strain is 1370 bp. A deletion of 9597 and 8309 bp is observed for the C1N1d strain relative to the R and S strains, respectively (dashed lines extending from the bottom to the top panels). The deletion in the C1N1d strain includes all of the coding exons of both *HR96-LBD-1a* and *HR96-LBD-1b*, as well as the gene *tetur06g04280* of unknown function with no homology to hormone receptor genes that is present in the London reference genome annotation. Manual annotation of *tetur06g04280* in the R and S strains revealed that it is likely a pseudogene in both strains as indicated (gray shading, see legend).

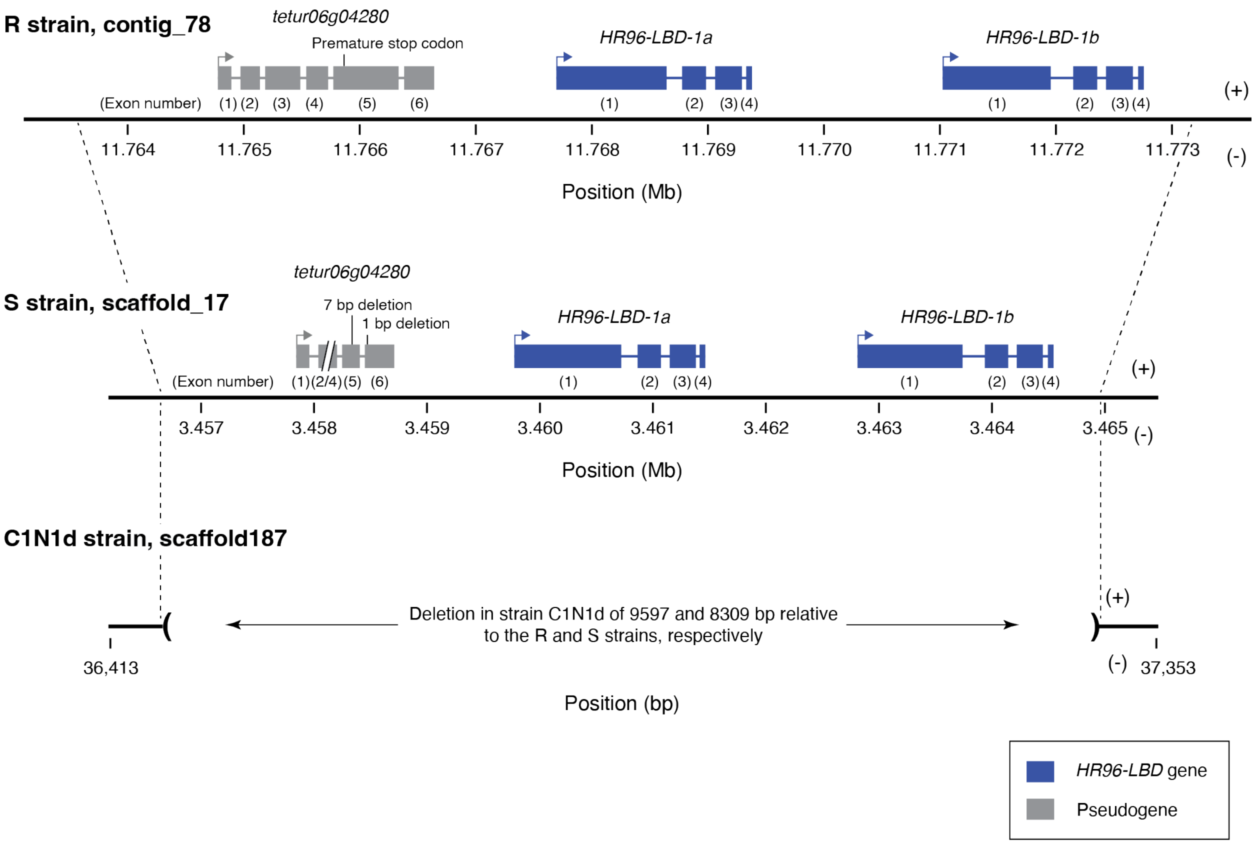

Supplementary Figure 9: Alignment of HR96-LBD-1a and HR96-LBD-1b sequences from the R and S strains.

Multiple sequence alignment of HR96-LBD-1a and HR96-LBD-1b amino acid sequences from the R and S strains. Shared sequence differences between the R and S strains between the two tandemly duplicated genes are indicated by green shading, while singleton changes are shaded in yellow (see legend, top). Invariant amino acids are denoted by "*", gaps by "-", conservative amino acid replacements by "." and ":", and non-conservative changes by empty spaces. The ligand-binding domain (LBD) is indicated above the aligned sequences (orange box labelled "LBD"); an arrow points to a radical W309R change (the coordinate is based on the HR96-LBD-1b sequence of the R strain). This figure is based on the sequence data provided in Supplementary Data 13.

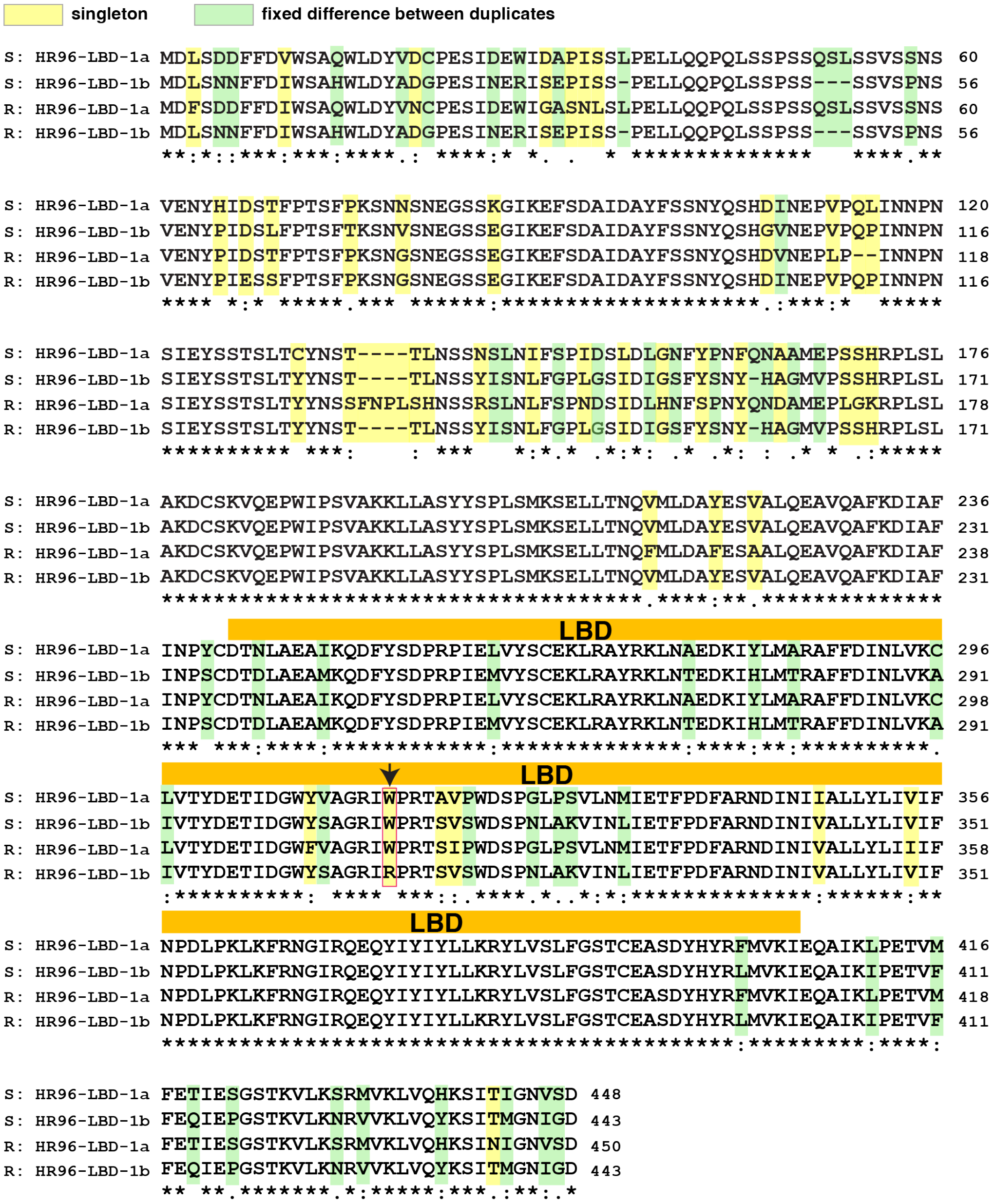

Supplementary Figure 10: Expression of *tetur86g00030* on bean and tomato following injections with dsGFP and dsHR96-LBD-1.

The expression of *tetur86g00030*, the closest potential dsHR96-LBD-1 RNAi off-target, was quantified by RT-qPCR after injection of dsGFP (control) and dsHR96-LBD-1 (treatment). Following the injections, mites were maintained for four days on bean ('bean' experiment) or mites were maintained on bean for three days before being transferred to tomato for 24 hours ('tomato' experiment). Expression levels are scaled to the mean of the control in each experiment. For the bar plot, means, error bars of ±1 standard deviation, and all data points are shown. Statistical insignificances (adj-$p > 0.05$; n.s., not significant; $n = 3$ biologically independent replicates, with each biological replicate based on two technical replicates) were observed with two-sided unpaired t-tests with correction for multiple tests (for mites reared on bean and tomato, respectively: $t(3.98) = 2.98$, $p = 0.08$; and $t(2.11) = 0.37$, $p = 0.75$; the p values are adjusted for multiple tests with the Benjamini-Hochberg method).

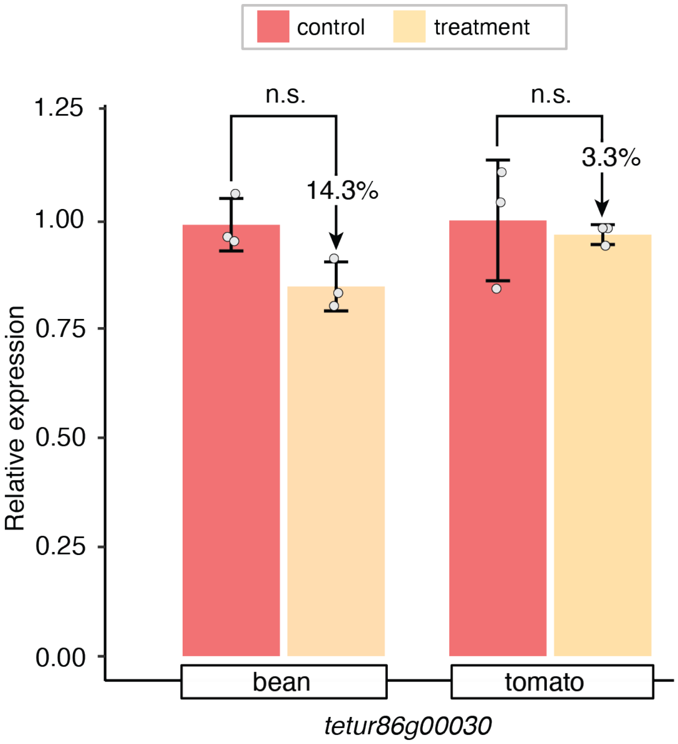

Supplementary Figure 11: The W309R strain variant in HR96-LBD-1b is predicted to reside in the ligand-binding pocket.

A sequence coverage plot with the multiple sequence alignment based on HR96-LBD-1b, generated by ColabFold, that was used as input for the AlphaFold2 network is shown in the top left panel, and the predicted lDDT (distance difference tests, a measure of confidence, also generated by ColabFold) per residue position is shown in the top right panel; these per position analyses revealed that only the region of amino acid residues from approximately 251 to 444 of HR96-LBD-1b were predicted with high confidence. The bottom panel displays a partial alignment of *T. urticae* HR96-LBD-1a and HR96-LBD-1b and related receptors of invertebrates or vertebrates with known crystal structures spanning residues 251 to 444 of HR96-LBD-1b that shows helices H3 to H12 (see Methods). The residues in the β-strand are colored in purple. The inside facing amino acids of the vertebrate PXR xenosensor ligand binding pocket, F281, F288, W299 and Y306, are indicated in blue. Note that the Y306 position in PXR is at the same location in the alignment as position 309 in *T. urticae* HR96-LBD-1b (bold black with red box for HR96-LBD-1a and HR96-LBD-1b), and among all sequences in the alignment is unique in that it is positively charged. While the W309R substitution in HR96-LBD-1b may impact signaling by altering ligand binding, or possibly by inducing a conformational change leading to constitutive activation (see Discussion), the dimer interface of the receptors is formed by helixes H7, H9 and H10, and the activation domain AF-2 is formed by H12 (residues in green), and therefore the W309R change is unlikely to impact the dimerization and activation potential of HR96-LBD-1b. Species: *Strongyloides stercoralis* (*S. stercoralis*), *Drosophila melanogaster* (*D. melanogaster*), and *Homo sapiens* (*H. sapiens*).

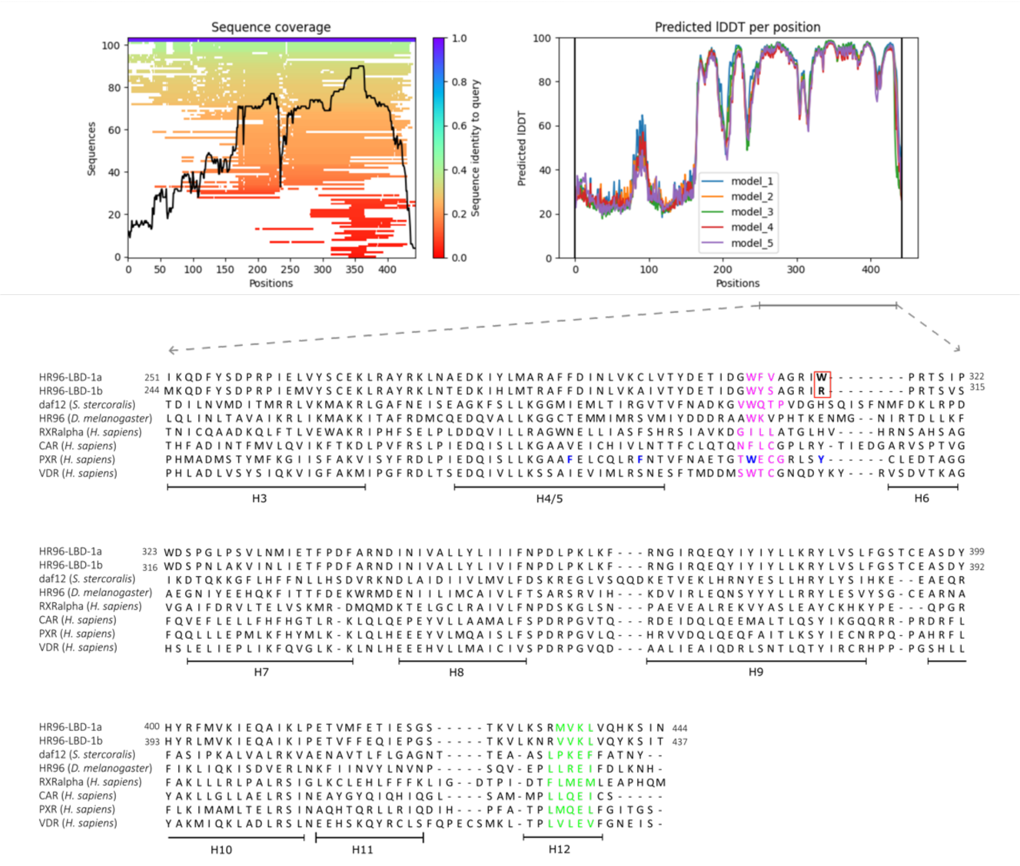

Supplementary Figure 12: Nucleotide variation in *T. urticae* strains resulting in the W309R amino acid change.

Alignments of DNA sequences for seven *T. urticae* strains at and nearby the first base in codon 309 of *HR96-LBD-1b* (position 0 in the alignments, bottom; the respective alignments for *HR96-LBD-1a* are shown at top). DNA sequences are shown for the forward strand relative to the direction of transcription. Bases with no variation across strains and the *HR96-LBD-1a* and *HR96-LBD-1b* duplicate genes are indicated in gray, and variable bases are in bold and are color-coded by nucleotide. In the 50 bp shown, six nucleotides fixed between *HR96-LBD-1a* and *HR96-LBD-1b* were used to assign sequences to the respective duplicate genes (denoted by asterisks at top). In *HR96-LBD-1b*, a change from T to C in strains R and RB underlies the amino acid W309R substitution (a change from codon TGG to CGG as indicated), while in strains MAR-ABi, Hib, KH, and WG-S an independent mutational event(s) resulted in the W309R substitution (a T-to-A transversion; TGG to AGG). Strains and the method of sequence ascertainment are indicated at left (see "Strain" and "Method" and see the Methods section for additional information; where the same sequence was obtained with independent methods, only one sequence is displayed). For instance, for strains S, R, and MAR-ABi identical sequences were obtained using either three or two independent methods of sequence collection (e.g., PacBio or Illumina assemblies, or duplicate-specific PCR followed by Sanger sequencing, "PCR+Sanger"). Strain S, as well as 14 other inbred strains that are not shown but that we included in our analyses, are inferred to have the ancestral T nucleotide at position 0 in the alignment in *HR96-LBD-1b*, with all strains having the T nucleotide at the respective position in *HR96-LBD-1a* (see Supplementary Data 12).

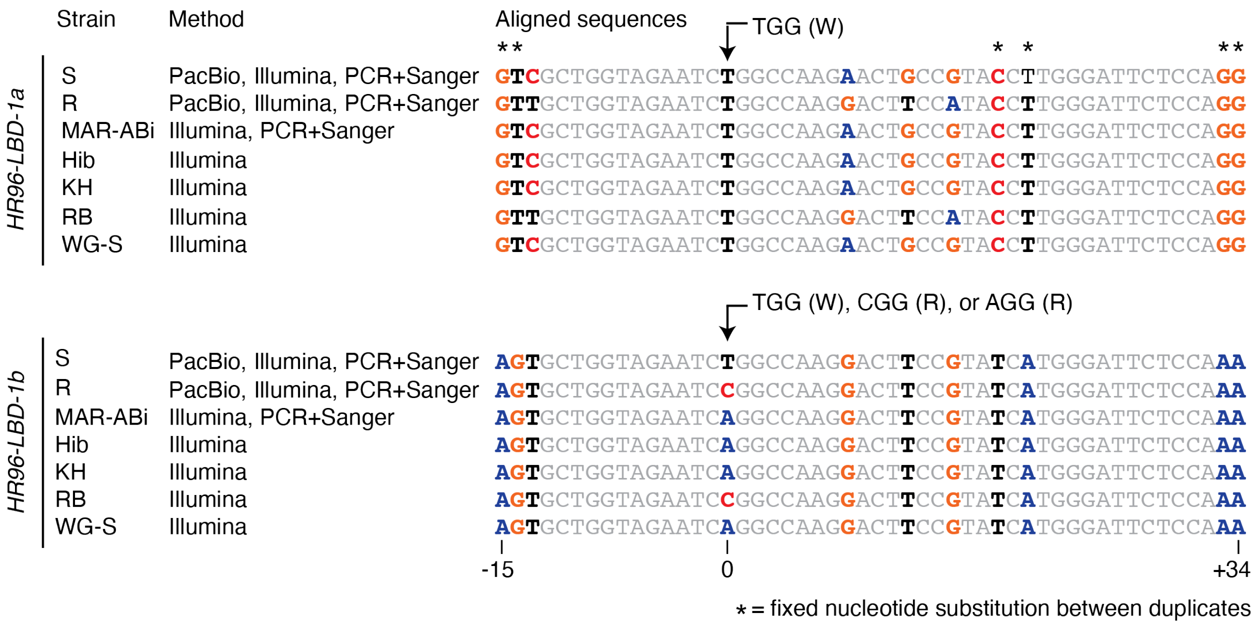

Supplement: Supplementary file 1 — Supplementary information [file 41467_2023_40778_MOESM1_ESM.pdf]
