# Supplementary material for: A nuclear receptor HR96-related gene underlies large trans-driven differences in detoxification gene expression in a generalist herbivore
Source: Nat Commun. 2023 Aug 17;14:4990. doi: 10.1038/s41467-023-40778-w (PMC10435515; doi:10.1038/s41467-023-40778-w)
Supplement: Supplementary file 2 — Reporting Summary [file 41467_2023_40778_MOESM2_ESM.pdf]

nature portfolio

Corresponding author(s): Richard Michael Clark; Thomas Van Leeuwen

Last updated by author(s): Aug 4, 2023

Reporting Summary

Nature Portfolio wishes to improve the reproducibility of the work that we publish. This form provides structure for consistency and transparency in reporting. For further information on Nature Portfolio policies, see our Editorial Policies and the Editorial Policy Checklist.

Statistics

For all statistical analyses, confirm that the following items are present in the figure legend, table legend, main text, or Methods section.

n/a	Confirmed	
☐	☒	The exact sample size (*n*) for each experimental group/condition, given as a discrete number and unit of measurement
☐	☒	A statement on whether measurements were taken from distinct samples or whether the same sample was measured repeatedly
☐	☒	The statistical test(s) used AND whether they are one- or two-sided<br>*Only common tests should be described solely by name; describe more complex techniques in the Methods section.*
☒	☐	A description of all covariates tested
☐	☒	A description of any assumptions or corrections, such as tests of normality and adjustment for multiple comparisons
☐	☒	A full description of the statistical parameters including central tendency (e.g. means) or other basic estimates (e.g. regression coefficient) AND variation (e.g. standard deviation) or associated estimates of uncertainty (e.g. confidence intervals)
☐	☒	For null hypothesis testing, the test statistic (e.g. *F*, *t*, *r*) with confidence intervals, effect sizes, degrees of freedom and *P* value noted<br>*Give P values as exact values whenever suitable.*
☒	☐	For Bayesian analysis, information on the choice of priors and Markov chain Monte Carlo settings
☒	☐	For hierarchical and complex designs, identification of the appropriate level for tests and full reporting of outcomes
☐	☒	Estimates of effect sizes (e.g. Cohen's *d*, Pearson's *r*), indicating how they were calculated

Our web collection on statistics for biologists contains articles on many of the points above.

Software and code

Policy information about availability of computer code

Data collection	None.
Data analysis	Scripts used in the data analysis, including information about programming languages and respective packages, are available at: https://github.com/rmclarklab/mite_eQTL; https://doi.org/10.5281/zenodo.7992545.<br>SNPs were called using GATK (v4.2);<br>DNA-seq alignments were performed with BWA (v0.7.17-r1188);<br>RNA-seq alignments were performed with STAR (v2.7.3a);<br>BAM files were sorted and indexed with SAMtools (v1.9);<br>Genotyping of population samples with aligned RNAseq reads used the Pysam package in Python 3.0 (v0.15.0);<br>Read-counts per gene were assessed with htseq-count (v2.0.1);<br>Gene domain predictions were performed with InterProScan (v91.0);<br>Multiple sequence alignments were performed with MAFFT (v7.505), except for those shown in Supplementary Fig. 11, which used PROTMALS3D (http://prodata.swmed.edu/ promals3d accessed online on 1 May, 2023, running PROTMALS3D v1);<br>Genome assemblies with PacBio reads were generated with Flye (v2.5);<br>Polishing of PacBio genome assemblies was performed with Pilon (v1.22);<br>The C1N1d genome assembly was performed with SOAPdenovo2 (v2.4);<br>Homology modeling of HR96-LBD-1b was performed with ColabFold (v1.5.039);<br>Statistical and other data analyses or visualizations were performed with R v4.1, including with R packages: MatrixEQTL (v2.3) for eQTL mapping, R/qtl (v1.46) for calculating recombination fraction (rf) and logarithm of the odds (LOD) scores, clusterProfiler (v4.2.2) for gene ontology enrichment analyses; DESeq2 (v1.34.0) was used for library size normalization for eQTL mapping and for differential gene expression

analyses, ComplexHeatmap (v2.10) for generation of a heatmap with expression data, VennDiagram (v1.7) for producing Venn diagrams, and ggplot2 (v3.3) for the generation of other display items;
Homology searches with HR96-LBD-1 were performed with BLAST (v2.6.0+); those for genes flanking the HR96-LBD-1 locus in C1N1d were performed using BLAST (v2.9.0+);
Manual gene annotation was performed using the GenomeView (vN42) environment;
Analysis of raw quantification cycle (Cq) values, including normalization against the housekeeping genes was performed with qbase+ (v5.1.17.0);
Detection of copy number variation used Python 3 and Pysam (v0.15.0);
Primer pairs were designed using Primer3 (v4.1.0);
Primer selection in order to minimize off-target effects in RNAi experiments was done using si-Fi (v21_1.2.3-0008);
Figures were adjusted as needed with Adobe Illustrator (v27.1.1).

For manuscripts utilizing custom algorithms or software that are central to the research but not yet described in published literature, software must be made available to editors and reviewers. We strongly encourage code deposition in a community repository (e.g. GitHub). See the Nature Portfolio guidelines for submitting code & software for further information.

Data

Policy information about availability of data

All manuscripts must include a data availability statement. This statement should provide the following information, where applicable:

- Accession codes, unique identifiers, or web links for publicly available datasets
- A description of any restrictions on data availability
- For clinical datasets or third party data, please ensure that the statement adheres to our policy

Data supporting the findings of this work, including eQTL mapping and differential gene expression analyses, are available within the paper and its Supplementary Information files. RNA-seq reads and gene expression metadata have been deposited at Gene Expression Omnibus (Project GSE221677). The S (genome version JAPRAR000000000) and R (genome version JAPRAS000000000) assemblies, along with the respective PacBio DNA reads, have been deposited to National Center for Biotechnology Information, NCBI (BioProjects PRJNA907360 and PRJNA907031, respectively). The C1N1d strain genome (genome version JASKHX000000000) assembly has been deposited to NCBI under the previously published BioProject PRJNA597924. Sanger sequences and targeted Illumina assemblies of HR96-LBD-1a and HR96-LBD-1b have been deposited at GenBank (accessions OR067932 to OR067949) and genetic marker data used for eQTL mapping are provided on FigShare (ref. 92). The assembly of the C1N1d strain used previously published Illumina DNA read data (NCBI BioProject PRJNA597924), and previously published Illumina DNA read data were also used for HR96-LBD-1a and HR96-LBD-1b copy number analyses (NCBI BioProjects PRJNA387043, PRJNA498683, PRJNA530192, PRJNA597924, and PRJNA799176) and for targeted de novo assemblies (NCBI BioProjects PRJNA530192 and PRJNA799176). RNA-seq data used for expression studies with the S and R strains were published previously (NCBI PRJNA801103). Previously published protein sequences, or structures, that supported HR96-LBD-1 alignments included NP_524493.1 (NCBI), pdb_6hn6 (Protein Data Bank, PDB), pdb_1XV9 (PDB), pdb_6XP9 (PDB), pdb_1DB1 (PDB), and pdb_3GYU (PDB). Source data are provided.

Human research participants

Policy information about studies involving human research participants and Sex and Gender in Research.

Reporting on sex and gender	NA
Population characteristics	NA
Recruitment	NA
Ethics oversight	NA

Note that full information on the approval of the study protocol must also be provided in the manuscript.

Field-specific reporting

Please select the one below that is the best fit for your research. If you are not sure, read the appropriate sections before making your selection.

[x] Life sciences   [ ] Behavioural & social sciences   [ ] Ecological, evolutionary & environmental sciences

For a reference copy of the document with all sections, see nature.com/documents/nr-reporting-summary-flat.pdf

Life sciences study design

All studies must disclose on these points even when the disclosure is negative.

Sample size

For the eQTL mapping experiment, 458 RNA samples were used to assure sufficient statistical power to detect eQTLs. The sample size of 458 was selected because it is about twice that of some other conceptually similar studies that successfully identified both trans and cis eQTLs in other organisms (for example: West et al. 2007. Global eQTL Mapping Reveals the Complex Genetic Architecture of Transcript-Level Variation in Arabidopsis. Genetics, 175, 1441–1450). For differential expression analyses of RNA-seq data between the near-isogenic lines, and for RNAi studies, five or four biologically independent samples were used. This number of replicates was selected because three biologically

	independent replicates have typically been shown to be sufficient for robust detection of differentially expressed genes in related studies. Further, key results were validated by RT-qPCR that used three biologically independent samples (two technical replicates per biological replicate). Cross validation between experiments (e.g., eQTL mapping, differential gene expression based on RNA-seq for NIL and RNAi samples, as well as complementary RT-qPCR) revealed that the sample sizes were sufficient to detect differences in gene expression for the same genes in a highly replicable manner across the different experiments used in the study.
Data exclusions	Only biological replicates with coefficient values of R2 > 0.9 were included in pairwise differential gene expression comparisons using near-isogenic lines at HS1. As described in the Methods section, a single B-NIL-HS1(RR) replicate that failed to satisfy this threshold was removed from the differential gene expression analyses.
Replication	Near isogenic lines of HS1 were produced in two independent biological replicates (i.e., originating from two different parental crosses). RNA-seq data for each set of lines, as well as for their matching F1 populations, was generated with 5-fold or 4-fold independent biological replication. Genes detected in the respective differential gene expression analyses replicated.<br><br>Near isogenic lines of CYP392A12 were produced in two independent biological replicates (i.e., originating from two different parental crosses). To understand the impact of the R genotype at HS1 on CYP392A12 expression, we crossed males for the NILs for CYP392A12 to B-NIL-HS1RR and B-NIL-HS1SS females with three biological replicates per cross and determined expression of CYP392A12 by RT-qPCR (two technical replicates each per biological replicate for RT-qPCR). Both experiments revealed that the RS genotype at both HS1 and CYP392A12 was required for elevated expression of CYP392A12 as compared to the S strain (that is, the finding was replicated).<br><br>RNAi experiments on bean were conducted with 5-fold biologically independent replication, while RNAi experiments on tomato were conducted with 4-fold biologically independent replication. A comparison between the two experiments revealed that in both the key genes controlled in trans by HS1 had lower expression in the treatment RNAi samples (that is, the findings replicated). Further, RT-qPCR on a subset of genes encoding HR96-LBD proteins was also performed, and replicated the findings of the respective RNA-seq experiments for the genes tested.
Randomization	For the eQTL mapping experiment, 458 F2 males derived from crossing two inbred lines were randomly sampled to created pools of isogenic females (that is, they were sampled before their genotypes were known).<br>Samples of near-isogenic lines were allocated to groups based on their genetic background.<br>Samples of F1 populations were allocated to groups based on the specific cross they originated from.<br>Samples of RNAi experiments were allocated to groups based on the treatment they received (i.e., injections with dsRNA against HR96-LBD-1 (treatment) or GFP (control)) and the host (i.e. bean or tomato) they were collected from.
Blinding	Blinding was not relevant for the eQTL mapping experiment (the collection of RNA-seq data by sample F3 family) as the genotype of the males that were sampled could only be inferred from the RNA-seq data following the completion of the laboratory work to generate the sample populations and the RNA used for production of RNA-seq data. For the respective studies with NILs, RNAi, and RT-pPCR, the small number of individuals (authors) involved in the study precluded blinding; however, in some cases this was not relevant or even possible (i.e., when adding mites to either bean or tomato leaves, the structure of the leaves makes them uniquely distinguishable to the investigator, and in fact this was used to ensure that no sample mix ups took place).

Reporting for specific materials, systems and methods

We require information from authors about some types of materials, experimental systems and methods used in many studies. Here, indicate whether each material, system or method listed is relevant to your study. If you are not sure if a list item applies to your research, read the appropriate section before selecting a response.

Materials & experimental systems

n/a	Involved in the study	
x		Antibodies
x		Eukaryotic cell lines
x		Palaeontology and archaeology
	x	Animals and other organisms
x		Clinical data
x		Dual use research of concern

Methods

n/a	Involved in the study	
x		ChIP-seq
x		Flow cytometry
x		MRI-based neuroimaging

Animals and other research organisms

Policy information about studies involving animals; ARRIVE guidelines recommended for reporting animal research, and Sex and Gender in Research

Laboratory animals	The mite Tetranychus urticae (the two-spotted spider mite).
Wild animals	This study did not involve wild animals.
Reporting on sex	Tetranychus urticae has a haplodiploid reproduction system; males are haploid, and females are diploid. Distinguishing adult males from females is easily done via visual morphological differences in adult mites (the only stage used in this study). T. urticae's

	haplodiploid reproductive system enabled us to set up specific crossing schemes that generated female sample famlies for eQTL mapping, and for follow-up studies with near-isogenic lines for validation purposes. All RNA samples originated from females.
Field-collected samples	This study did not use field-collected samples.
Ethics oversight	No ethical approval was needed as this work is conducted with invertebrates.

Note that full information on the approval of the study protocol must also be provided in the manuscript.
