# Supplementary material for: A nuclear receptor HR96-related gene underlies large trans-driven differences in detoxification gene expression in a generalist herbivore
Source: Nat Commun. 2023 Aug 17;14:4990. doi: 10.1038/s41467-023-40778-w (PMC10435515; doi:10.1038/s41467-023-40778-w)
Supplement: Supplementary file 4 — Description of Additional Supplementary Files [file 41467_2023_40778_MOESM4_ESM.pdf]

Description of Additional Supplementary Files

File Name: Supplementary Data 1
Description: Genotype compositions for the 458 F3 isogenic families used for eQTL mapping (columns "library" and "family name") based on RNA-seq data. There are two possible genotypes, either heterozygous RS or homozygous SS, for any genomic interval for which the range is indicated by columns "chromosome", "start", and "end". The "left SNP" and "right SNP" columns indicate the positions of the SNPs used for assigning the respective value in the "end" column in any row. Entries for the "left SNP" and "right SNP" columns are "-" for the final genotype blocks along chromosomes (or alternatively if an entire chromosome was assigned to a single genotype).

File Name: Supplementary Data 2
Description: Chromosome, beginning position (bp), ending position (bp), and marker position (bin midpoints, bp) for genotype bins used for eQTL mapping (columns "chromosome", "beginning of genotype bin (bp)", "ending of genotype bin (bp)", and "marker (bin midpoint, bp)", respectively).

File Name: Supplementary Data 3
Description: Significant associations between genes and respective eQTLs (adjusted-$p < 0.01$; the p-values are from MatrixEQTL based on the "modelLINEAR" option and were corrected for multiple tests in MatrixEQTL). There are three types of eQTLs (column "eQTL_label"): *cis* (gene start position is < ±800 kb from the midpoint of the eQTL genotype bin), *trans* (gene start position is > ±800 kb from the midpoint of the eQTL genotype bin), and eQTL (for a gene on a small scaffold where *cis* or *trans* could not be assigned using distance criteria). The chromosome and coordinates of genes are given in columns "gene_chromosome", "gene_start", and "gene_end". The effect sizes for eQTLs on target genes is indicated in column "beta" based on a linear model. The column "detox label" indicates the detoxification family, with gene names, where available, in parentheses (entries for non-detoxification genes are empty). Gene descriptions are given in column "gene_description".

File Name: Supplementary Data 4
Description: Summary information (the left-most column) for eQTL for all genes (column "all genes") and genes in detoxification gene families (column "detoxification genes"). Sample size: n (for genes with eQTLs). For columns "all genes" and "detoxification genes" both the sample size and percentages (in parentheses) are provided.

File Name: Supplementary Data 5
Description: Gene membership in detoxification gene families. For each gene, its family, name (if available), gene coordinates (columns "chromosome", "start", and "end"), and gene description are provided.

File Name: Supplementary Data 6
Description: Genes regulated by *trans* eQTLs overlapping the *trans* eQTL hotspots (HS1-HS9). For each *trans* eQTL hotspot, the number of target genes (n) and detoxification genes (detox), along with the range of the hotspot interval, are indicated in column "HS". For each *trans*-regulated gene, its *trans* eQTL location is indicated in the "eQTL (genotype bin)" column, and gene coordinates (columns "gene_chromosome", "gene_start", and "gene_end") are provided. The effect sizes from MatrixEQTL of the eQTLs on target genes is indicated in the "beta" column, and the significance of associations are indicated in the "adjusted-p" column (the p-values are from MatrixEQTL based on the "modelLINEAR" option and were corrected for multiple tests in MatrixEQTL). The columns "mean exp SS" and "mean expression RS" provide the expression means for the SS and RS genotypes at the respective *trans* eQTLs based on the 458 families of the eQTL mapping population (means were calculated based on DESeq2 normalized values). Column "log_2FC (RS vs. SS)" provides the respective log_2 fold change values between "mean expression RS" and

"mean expression SS". The column "detox label" indicates the detoxification family, with gene names, where available, in parentheses (entries for non-detoxification genes are empty). Gene descriptions are given in column "gene_description".

File Name: Supplementary Data 7
Description: For the nine *trans* eQTL hotspots (HS1-HS9, column "hotspot") with more than 100 *trans*-regulated genes, the genes located within genotype bins with midpoints overlapping the hotspot intervals are provided (column "genes in genotype bins overlapping hotspot"). The position of each hotspot (chromosome and position in Mb) is provided in column "hotspot region". For all genes, positions and descriptions (where available) are provided in columns "gene position (bp)" and "gene description", respectively.

File Name: Supplementary Data 8
Description: Gene ontology (GO; based on molecular function) enrichment analyses for *trans*-regulated genes by each of the nine hotspots (column "hotspot"; HS1-HS9). For each significantly enriched GO term (adjusted-$p < 0.05$; column "GO term"), its respective description, gene ratio, background gene ratio, adjusted-p value, and genes with the GO term are provided in columns "description", "GeneRatio", "BgRatio", and "p.adjust", and "geneID", respectively. The effect sizes of respective *trans* eQTLs on target genes is indicated in the "beta" column (based on a linear model). The column "detox label" indicates the detoxification family, with gene names, where available, in parentheses (entries for non-detoxification genes are empty). Gene descriptions are given in column "gene_description". The values in the p.adjust column are p-values from hypergeometric tests (one-sided tests) with the Benjamini-Hochberg adjustment for multiple tests (as performed with clusterProfiler).

File Name: Supplementary Data 9
Description: Genes located in the 200 kb HS1 window (column "HS1 interval", shaded light yellow) and in the surrounding regions for which R haplotypes were introgressed into A-NIL-HS1^{RR} or B-NIL-HS1^{RR}. Genotypes for A-NIL-HS1^{RR} and B-NIL-HS1^{RR} at given gene locations are indicated in the "A-NIL-HS1(RR) genotype" and "B-NIL-HS1(RR) genotype" columns (the two possible genotypes of RR and SS; the RR introgressed regions are shaded yellow). Gene coordinates are provided in the "chromosome", "start", and "end" columns. Gene strand is indicated by "+" (forward) or "-" (reverse) in column "strand". Gene descriptions are given in column "gene_description".

File Name: Supplementary Data 10
Description: Output of DESeq2 for pairwise comparisons to identify differentially expressed genes between NILs with and without the R haplotype introgression at HS1 (A-NIL-HS1^{RR} versus A-NIL-HS1^{SS}, and B-NIL-HS1^{RR} versus B-NIL-HS1^{SS}). Output for pairwise comparisons between F1s from NIL-HS1^{RR} × NIL-HS1^{SS} (A-NIL-HS1^{F1}, B-NIL-HS1^{F1}) and each of the respective parents are also provided. For each pairwise comparison, the DESeq2 calculated $\log_2$ fold change (column "$\log_2$FC"), standard error for $\log_2$FC (column "lfcSE"), and adjusted-p (column "padj") values are provided for each gene (the values in the padj columns are adjusted for multliple tests by DESeq2). The column "detox label" indicates the detoxification family, with gene names, where available, in parentheses (entries for non-detoxification genes are empty). Gene descriptions are given in column "gene_description".

File Name: Supplementary Data 11
Description: Genes with *trans*-eQTL at HS1 (column "HS1 (eQTL, RS vs. SS)"), differentially expressed genes in pairwise HS1 NIL comparisons (columns "A-NIL-HS1(RR) vs. A-NIL-HS1(SS)" and "B-NIL-HS1(RR) vs. B-NIL-HS1(SS)"), and differentially expressed genes in RNAi treatment versus control comparisons on bean and tomato (columns "RNAi (treatment vs. control, on bean)" and "RNAi (treatment vs. control, on tomato)", respectively) are as indicated. For RNAi samples, treatment represents dsHR96-LBD-1 injection, and control represents dsGFP injection, of B-NIL-HS1(RR) mites. The direction of gene

expression change (labels "up" or "down") is based on the pairwise comparisons as indicated in the header line (where no association or differential expression was observed, cells are empty). Numbers in brackets are the log2FC values associated with the eQTLs at HS1 (effects on target genes), and the log2FC values for NILs and RNAi from the pairwise or treatment-control comparisons as indicated. Gene coordinates are provided in columns "chromosome", "start", and "end". The column "detox label" indicates the detoxification family, with gene names, where available, in parentheses (entries for non-detoxification genes are empty). Gene descriptions are given in column "gene_description".

File Name: Supplementary Data 12
Description: For 22 inbred strains, including the R (MR-VPi) and S (ROS-ITi) strains (column "strain"), the normalized coverage in Illumina DNA-seq reads aligned to the reference London genome sequence, which has one copy of the *HR96-LBD-1* gene, is provided for the the *HR96-LBD-1* coding sequence (column "normalized HR96-LBD-1 CDS coverage"; normalization was to the coverage depth for other coding sequences genome-wide, and a coverage of ~1.0 is expected for single copy genes, see Methods). At the codon that encodes the amino acid at position 309 in HR96-LBD-1b in the R strain (the analogous coordinates are 12494170-12494172 on chromosome 1 in the London reference genome sequence), the number of reads supporting nucleotide T (column "T (TGG → W, ancestral)"), C (column "C (CGG → R, derived)"), and A (column "A (AGG → R, derived)") and the respective normalized coverage at that site (in parenthesis) is provided (the respective impacts of the nucleotides on the codon and encoded amino acid are provided in the column names). All Illumina read data used for the analysis has been published previously (the NCBI BioProject and Accession numbers are provided in columns "bioproject no." and "accession no.", respectively; the number of bases (in Gb) for the respective samples are given in column "bases in sample (Gb)"). Strain collection information is provided in column "original collection information"; the sources of this and other information (including the respective BioProject and accession numbers) are provided in column "References for DNA-seq bioproject and accession identifiers, and additional information" (see also Methods). CDS: coding sequence.

File Name: Supplementary Data 13
Description: DNA and protein sequences encoded by *HR96-LBD-1a* and *HR96-LBD-1b* in the R and S strains. The contigs harboring *HR96-LBD-1a* and *HR96-LBD-1b* in the PacBio-based assemblies of the R and S strains, and the respective exon coordinates, and strandedness, are provided in columns "contig in PacBio assembly", "coordinates in contig", and "strand", respectively.

File Name: Supplementary Data 14
Description: Output of DESeq2 for a differential gene expression analysis between the R versus S strains using RNA-seq data. The base mean, $\log_2$ fold change ($\log_2$FC), standard error for $\log_2$FC, and adjusted-*p* values for each gene are provided in columns "baseMean", "$\log_2$FC", "lfcSE", and "padj", respectively (the values in the padj column are adjusted for multliple tests by DESeq2).

File Name: Supplementary Data 15
Description: Output of DESeq2 for pairwise comparisons to identify differentially expressed genes between mites injected with dsHR96-LBD-1 (treatment) versus dsGFP (control) feeding on bean or on tomato (column headers "treatment vs. control (on bean)" and "treatment vs. control (on tomato)"), and for control samples of mites feeding on tomato versus on bean (column "tomato vs. bean (control)"). For each comparison, $\log_2$ fold change ($\log_2$FC), standard error for $\log_2$FC, and adjusted-*p* values for each gene are provided in columns "$\log_2$FC", "lfcSE", and "padj", respectively (the values in the padj columns are adjusted for multliple tests by DESeq2).

File Name: Supplementary Data 16
Description: Gene set (column "gene set") classifications ("Set1", "Set2", or "other") for genes identified in treatment versus control RNAi comparisons. The column "detox label" indicates the detoxification

family, with gene names, where available, in parentheses (entries for non-detoxification genes are empty). Gene descriptions are given in column “gene_description”.

File Name: Supplementary Data 17
Description: Enriched gene ontology (GO, based on molecular function) terms for genes in Set1 and Set2. For each significantly enriched GO term (adjusted $p < 0.05$; column “GO term”), the respective description, gene ratio, background gene ratio, adjusted-*p* value, and genes with the term are provided in columns “description”, “GeneRatio”, “BgRatio”, “p.adjust”, and “geneID”, respectively. The column “detox label” indicates the detoxification family, with gene names, where available, in parentheses (entries for non-detoxification genes are empty). Gene descriptions are given in column “gene description”. The values in the p.adjust column are *p*-values from hypergeometric tests (one-sided tests), with the Benjamini-Hochberg adjustment for multiple tests as performed with clusterProfiler.

File Name: Supplementary Data 18
Description: For each of the 458 RNA-seq samples (one for each isogenic F3 family) used for eQTL mapping (column “library”), the number of resulting total paired-reads, uniquely mapped reads, and the percentage of uniquely mapped reads are provided in columns “total reads”, “uniquely mapped reads”, and “uniquely mapped (%)”, respectively.

File Name: Supplementary Data 19
Description: Cleaved Amplified Polymorphic Sequence (CAPS) markers (column “CAPS markers”) and their primers and respective Tm values (columns “forward sequence (5' → 3')”, “reverse sequence (5' → 3')”, “forward Tm (°C)”, and “reverse Tm (°C)”) used in the construction of near-isogenic lines (NIL) in the HS1 and *CYP392A12* genomic regions. The location and allelic state of variable sites about which CAPS markers were designed, and the respective restriction enzymes used, are given in columns “chromosome”, “position (bp)”, “S allele”, “R allele”, and “restriction enzyme (site)”. The lengths of S and R strain cleavage products are given in columns “S length (bp)” and “R length (bp)”, respectively.

File Name: Supplementary Data 20
Description: Primers used for RT-qPCR for genes listed in columns “gene” and “name”. The forward and reverse primer sequences, as well as the respective Tm values and product lengths, are given in columns “forward sequence (5' → 3')”, “reverse sequence (5' → 3')”, “forward Tm (°C)”, “reverse Tm (°C)”, and “S length (bp)”, respectively. Where applicable, references (with DOIs) are provided (column “reference”).

File Name: Supplementary Data 21
Description: Primers used for amplification of products for use in dsRNA production against *HR96-LBD-1* and GFP (column “target gene”). In the primer sequences (columns “forward sequence (5' → 3')” and “reverse sequence (5' → 3')”), lowercase letters are the T7 promoter sequence used for *in vitro* transcription. The respective primer Tm values and the product lengths are provided in columns “forward Tm (°C)”, “reverse Tm (°C)”, and “fragment length (bp)”.

File Name: Supplementary Data 22
Description: Primers used for the amplification of *HR96-LBD-1a* and *HR96-LBD-1b* (column “target gene”) including sequences, Tm values, and the expected amplicon length are provided (columns “forward sequence (5' → 3')”, “reverse sequence (5' → 3')”, “forward Tm (°C)”, “reverse Tm (°C)”, and “fragment length (bp)”, respectively). The strain specificity of primers is provided in column “strain specificity”.
